# Identification of adipocytes as target cells for *Leishmania infantum* parasites

**DOI:** 10.1038/s41598-021-00443-y

**Published:** 2021-10-28

**Authors:** Aurélie Schwing, Didier F. Pisani, Christelle Pomares, Alissa Majoor, Sandra Lacas-Gervais, Jennifer Jager, Emmanuel Lemichez, Pierre Marty, Laurent Boyer, Grégory Michel

**Affiliations:** 1 Université Côte d’Azur, CHU, Inserm, C3M, Nice, France; 2 Université Côte d’Azur, Inserm, C3M, Nice, France; 3 Université Aix-Marseille, Marseille, France; 4 Université Côte d’Azur, CNRS, LP2M, Nice, France; 5 Université Côte d’Azur, Centre Commun de Microscopie Appliquée, Nice, France; 6 Institut Pasteur, CNRS UMR2001, Unité des Toxines Bactériennes, 75015 Paris, France

**Keywords:** Parasite immune evasion, Parasitic infection

## Abstract

*Leishmania infantum* is the causative agent of visceral leishmaniasis transmitted by the bite of female sand flies. According to the WHO, the estimated annual incidence of leishmaniasis is one million new cases, resulting in 30,000 deaths per year. The recommended drugs for treating leishmaniasis include Amphotericin B. But over the course of the years, several cases of relapses have been documented. These relapses cast doubt on the efficiency of actual treatments and raise the question of potential persistence sites. Indeed, *Leishmania* has the ability to persist in humans for long periods of time and even after successful treatment. Several potential persistence sites have already been identified and named as safe targets. As adipose tissue has been proposed as a sanctuary of persistence for several pathogens, we investigated whether *Leishmania infantum* could be found in this tissue. We demonstrated both in cell cultures and in vivo that *Leishmania infantum* was able to infect adipocytes. Altogether our results suggest adipocytes as a ‘safe target’ for *Leishmania infantum* parasites.

## Introduction

*Leishmania infantum* (*L. infantum*) is the causative agent of visceral leishmaniasis and is transmitted by the bite of female sand flies. According to the WHO, the estimated annual incidence is one million new cases, resulting in 30,000 deaths per year^1^. The outcome of infection can be variable, ranging from an asymptomatic form in immunocompetent individuals to obvious disease^2^. These clinical features depend on the species and the immune response of the host^3^. Symptomatic leishmaniasis is characterized by 3 main forms: cutaneous (CL), mucocutaneous (MCL) and visceral leishmaniasis (VL). CL is a chronic infection with ulcerative skin lesion occurring at the site of inoculation. MCL is generally the result of parasite dissemination from the skin to the naso-oropharyngeal mucosa. VL is the most serious form of leishmaniasis and typically leads to death in a few months in the absence of treatment. It is characterized by irregular fever, weight loss, hepatosplenomegaly, lymphadenopathies and pancytopenia. Currently, there is no human vaccine and treatments are expensive, with WHO guidelines recommending the use of just a few drugs, such as Amphotericin B^4^. Over time, several cases of relapses have been documented^5,6^, which thus call into question the efficiency of current treatments^5,7^ and raise the unsolved question of host sites allowing parasite persistence. Indeed, *Leishmania* has the ability to persist in humans for long periods of time, even after successful treatment^8^. Several potential persistence sites have already been identified and named as safe targets. These include immature myeloid precursor cells, monocytes, sialoadhesin-positive stromal macrophages of the bone marrow, hepatocytes and fibroblasts^8^. Previously, *L. infantum* persistence and development has been demonstrated in intra-abdominal adipose tissue of intraperitoneally infected mice^9^. Moreover, adipose tissue has been proposed as a sanctuary of persistence for bacteria such as *Mycobacterium (M.) tuberculosis*^10^ or *M. canettii*^11^, *Coxiella burnetii*^12^, viruses such as Human or Simian Immunodeficiency Virus^13,14^, and parasites such as *Trypanosoma (T.) cruzi* or *T. brucei* and *Plasmodium* spp.^15,16^. Here, we address the issues of whether *L. infantum* infects adipose tissues and whether adipocytes represent host cells for these parasites.

## Results

### *L. infantum* is found in the adipose tissue of infected mice

**Figure 1:**
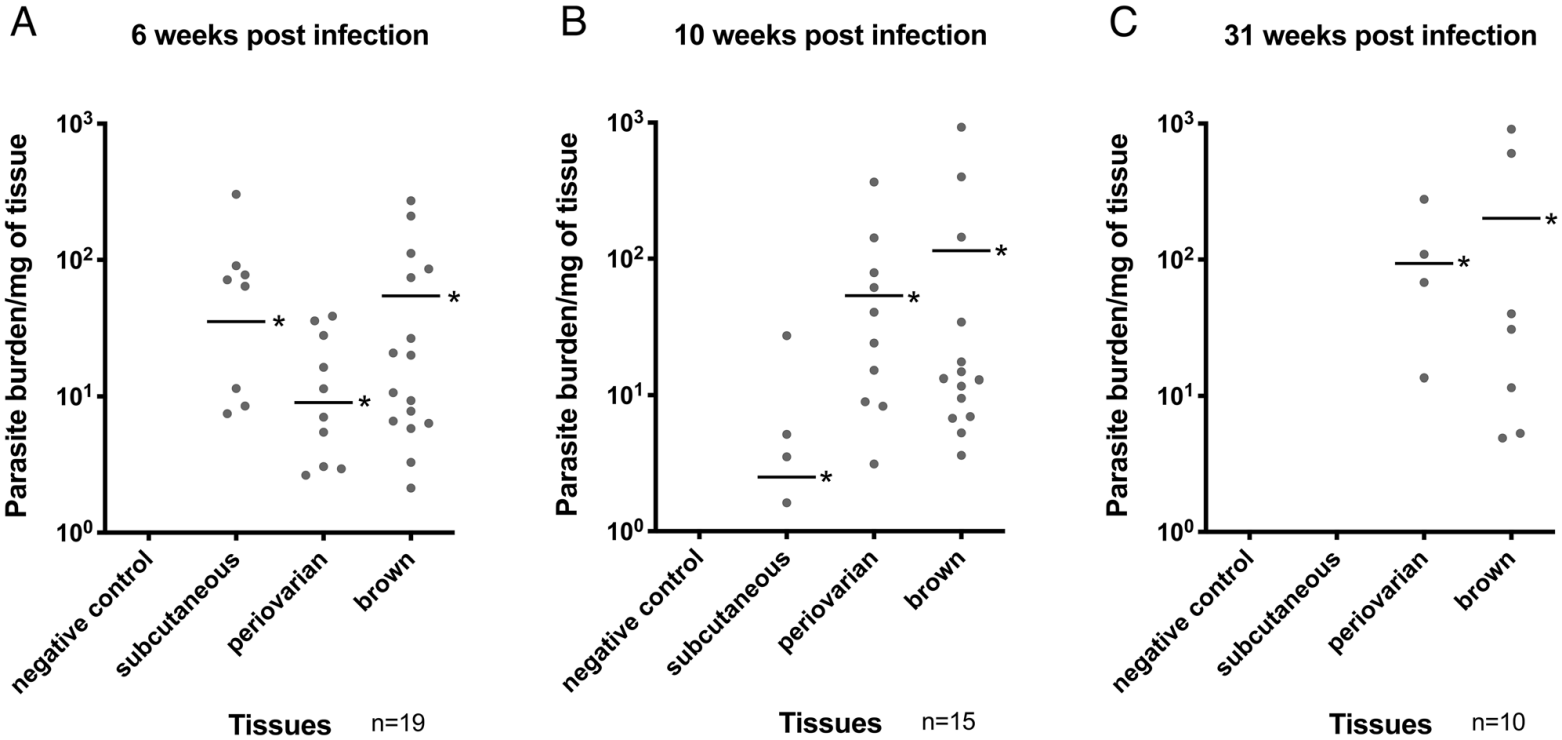
*L. infantum* parasites are present in adipose tissue from BALB/c mice. Subcutaneous, periovarian, and brown adipose tissue (25 mg) from mice inoculated with *L. infantum* were collected after different periods of time (6 (**A**), 10 (**B**) and 31 weeks post infection (**C**)), DNA was extracted in a 100 µL volume and the presence of *L. infantum* DNA was determined by qPCR using a 2.5 µL DNA extract. Results represent parasite burden per milligram of tissue with SEM. *p < 0.05. qPCR negative samples are not shown on graphs.

**Figure 2:**
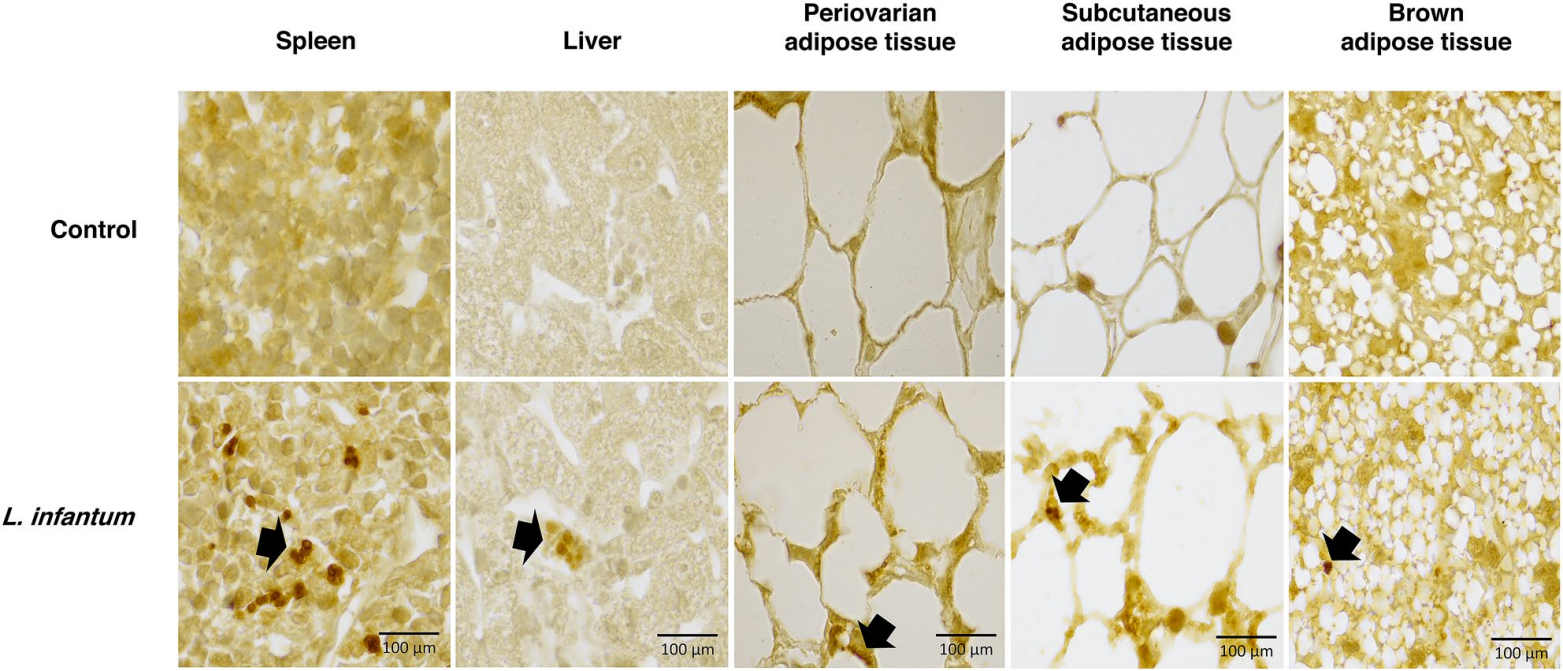
Immuno-labeled histological sections of tissues from BALB/c mice infected with *Leishmania infantum*, 40 weeks post infection. The presence of *Leishmania infantum* was determined in sections of different paraffin-embedded adipose tissue using rabbit anti-*Leishmania* polyclonal antibodies. Parasites were revealed using biotin-conjugated antibodies and peroxidase-labeled streptavidin. Labeled *Leishmania infantum* appear in orange/brown in adipocyte tissue (black arrows).

First, we investigated whether *L. infantum* was present in brown (BAT) and white (WAT) adipose tissue of infected mice. For this purpose, BALB/c mice were intravenously inoculated with LUC-*L. infantum* as previously described^9^. Although bioluminescent parasites were used in this study, parasite burden in adipose tissue was insufficient to obtain an exploitable signal and allow in vivo monitoring of infection. Mice were thus sacrificed 6, 10 and 31 weeks post infection (p.i.). Tissue samples were analyzed for the presence of *L. infantum* by qPCR, which confirmed chronic infection in the spleen and liver (Fig. S1). At 6 weeks p.i., subcutaneous and periovarian BAT and WAT presented parasite burden which remained high in BAT 10 weeks p.i. In subcutaneous tissue, between 6 and 10 weeks p.i., parasite burden decreased then totally disappeared after 31 weeks (Fig. 1). In contrast, the percentage of mice with positive parasite burden in BAT remained higher than 70%. These results indicated the persistence of *L. infantum* in BAT after infection by the intravenous route. Furthermore, we performed histological sections on different tissues from the same infected mice in order to visualize parasites by immunolabelling. 40 weeks p.i., the presence of *L. infantum* parasites in all types of adipose tissue could be observed (Fig. 2). This raised the question of whether parasites contained in adipose tissue were alive and endowed with the capacity to infect. We thus performed an adoptive transfer experiment by intravenously injecting naive mice with BAT homogenate from infected mice. 34 weeks post-transfer we were able to detect parasite DNA by qPCR in the liver, spleen and BAT of secondary-infected mice (Fig. S2). Taken together, our results show that *L. infantum* parasites present in the adipose tissue of mice kept their infectivity.

### *L. infantum* is present in adipocyte-enriched fractions

**Figure 3:**
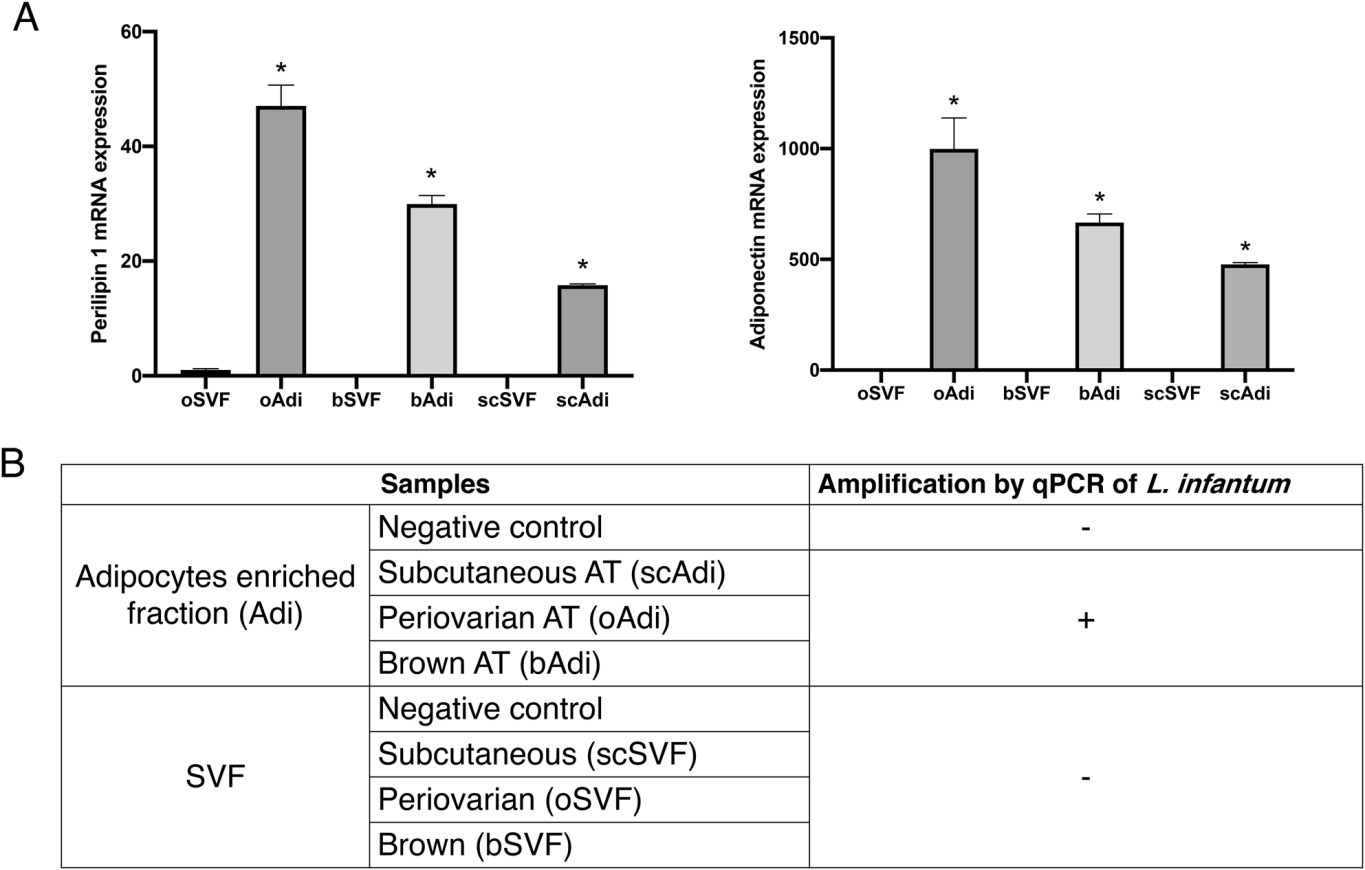
(**A**) mRNA expression of Perilipin 1 and Adiponectin determined by RT-qPCR in the stroma vascular fraction (SVF) and the adipocyte fraction from scAdi, oAdi and bAdi of BALB/c mice in brown and white adipocytes (subcutaneous and periovarian) and stromal vascular fractions. Perilipin-1 and Adiponectin mRNA expression were used as a control for adipocyte purification. Histograms represent mean + sem of 4 mice. (**B**) Detection by qPCR of *Leishmania infantum* in adipose tissue fractions. DNA was extracted in a 100 µL volume and the presence of *L. infantum* DNA was determined by qPCR using a 2.5 µL DNA extract. *p < 0.05.

To assess whether *L. infantum* was present in adipocytes or other cell types, we separated an adipocyte-enriched fraction from the stromal vascular fraction (SVF). WAT and BAT from 4 mice infected intravenously were isolated and floating fractions enriched in adipocytes were obtained. The samples were pooled in order to increase the number of parasites and improve detection by qPCR. Separation between adipocytes and stromal cells was checked by amplification of adipocyte marker mRNA (Fig. 3A). As expected, expression of perilipin 1, a protein coating the lipid droplet and abundantly expressed only in white and brown adipocytes^17^, Uncoupling Protein 1 (UCP-1) Mitochondrial protein responsible for thermogenic respiration, a specialized capacity of brown adipose tissue, as well as adiponectin, a glycoprotein adipocyte-specific factor^18^, were specifically detected in the adipocyte fraction. As no parasite DNA was detected in the SVF of each kind of adipose tissue (Fig. 3B), these results demonstrated the localization of *L. infantum* in adipocytes.

### *L. infantum* can infect both murine and human adipocytes in vitro

**Figure 4:**
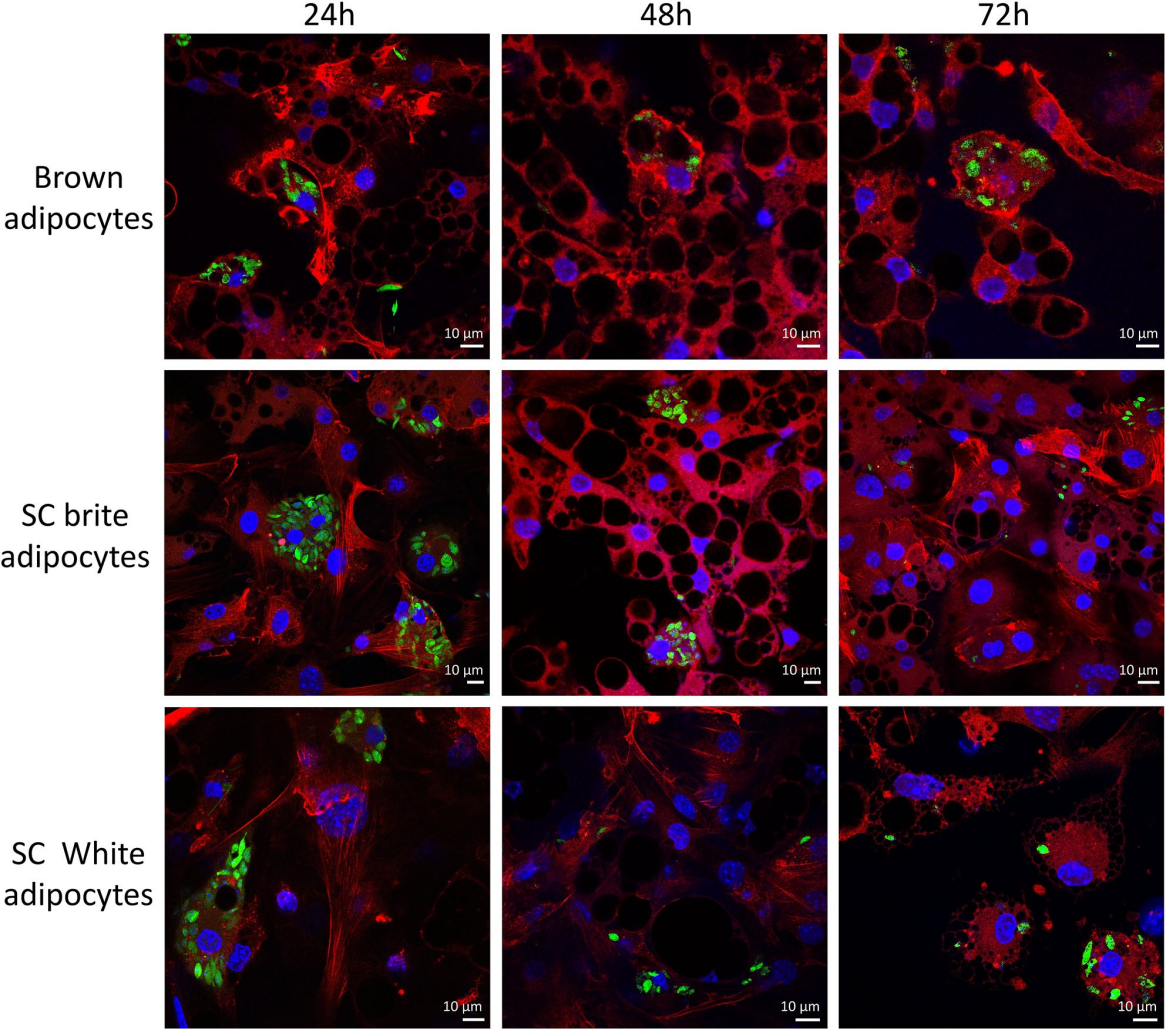
Confocal microscopy of BALB/C adipocytes infected In vitro with GFP-*L. infantum*. Adipocytes of pre-adipocyte murine origin were infected after 7 days of differentiation with 10 GFP-*Leishmania*/cells. Red: phallloidin-Txred, Blue: Dapi, Green: GFP-leish. Images were acquired with Nikon Confocal A1R software (NIS-Elements Confocal) from Nikon (https://www.microscope.healthcare.nikon.com). Images were merged with ImageJ bundled with Java 1.8.0_172 (https://imagej.nih.gov/ij/). Images were assembled with Adobe Photoshop 2020 (https://www.adobe.com/).

**Figure 5:**
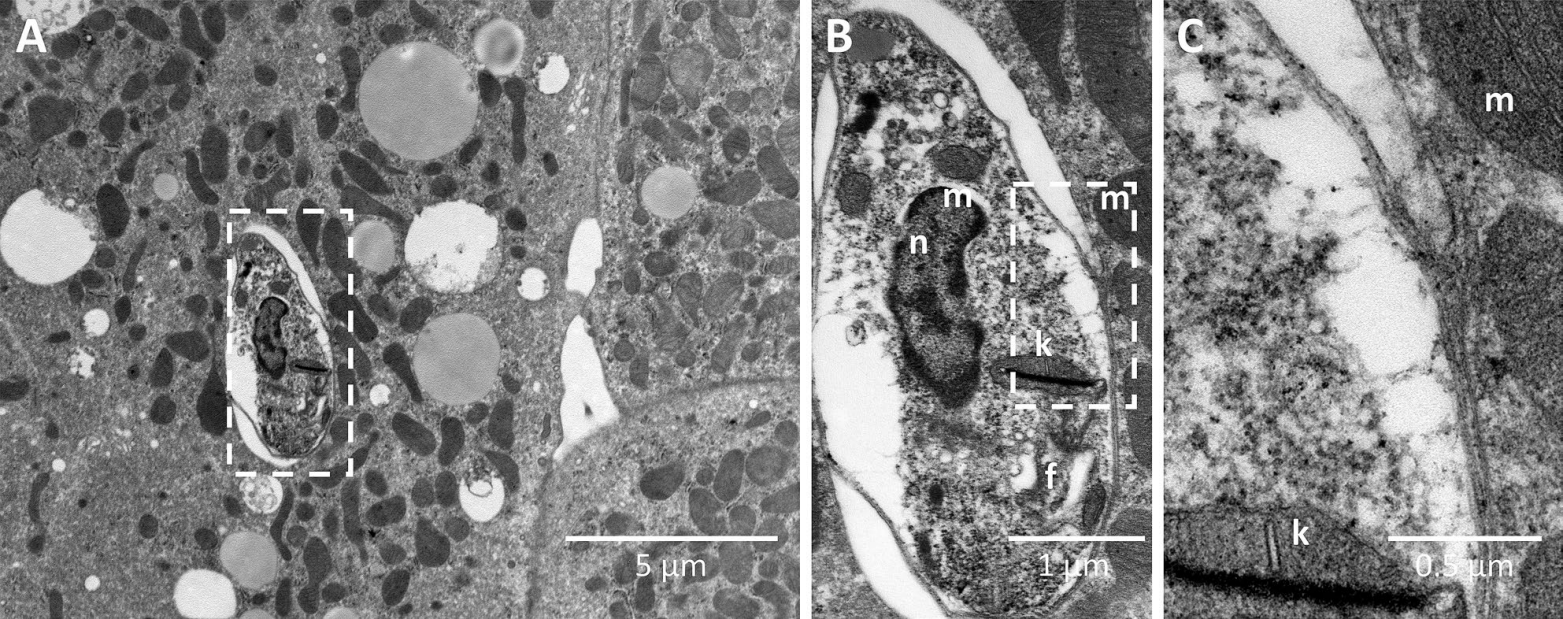
(**A–C**) Electron microscopy of in vitro infected brown adipocytes by GFP-*L. infantum* with different magnification (nucleus (n), flagella (f), kinetoplast (k), mitochondrion (m)).

We next evaluated in vitro, the ability of *L. infantum* to infect white, brown and brite adipocytes derived from primary mouse pre-adipocytes. Brite adipocytes are similar to classical brown adipocytes although they are derived from WAT. Primary pre-adipocytes from BALB/c mice were collected in subcutaneous (SC) white adipose tissue and differentiated into white and brite adipocytes. Pre-adipocytes from BAT were differentiated into brown adipocytes. 24, 48 and 72h post-infection, observation of the cells suggested the presence of intracellular parasites. This infection was observed in all types of adipocytes, and indicated the ability of *L. infantum* to infect and survive in murine adipocytes in vitro (Fig. 4). Given that *L. infantum* targets macrophages, bone marrow-derived macrophages (BMDM) were taken as a positive control for infection (Fig. S3). We subsequently observed the presence of parasites in human white and brite adipocytes differentiated from human adipose tissue stromal cells (Fig. S4)^19^. Specificity of *in vitro* differentiated adipocytes was assessed by qPCR for both adipocytes isolated from mouse and human donors (Fig. S5). To confirm the intracellular localization of *L. infantum* parasites in brown adipocytes, we performed an electron microscopy experiment. *L. infantum* parasites were indeed found inside a vacuole within lipid droplet-containing cells (Fig. 5). Moreover, using confocal microscopy we found GFP-*L.infantum* parasites inside 3T3-L1 adipocytes (Fig. S6). Altogether, by combining confocal and electron microscopy approaches, we confirmed the ability of *L. infantum* to parasitize adipocytes. Here we provide in vivo and in vitro evidence demonstrating that adipocytes are *bona fide* host cells for *L. infantum* parasites.

## Discussion

*Leishmania* persistence in humans is a critical medical problem but their sanctuaries remain undetermined. Adipose tissue has been recently hypothesized as a reservoir for several intracellular pathogens that are able to induce relapses^10–15^. Moreover, we previously highlighted the presence of *L. infantum* in intra-abdominal fat in BALB/c mice^9^. Here, we investigated whether or not adipocytes could be infected and if the adipose tissue could be a sanctuary of persistence for *L. infantum*.

Our results have shown the presence of *L. infantum* in murine subcutaneous, periovarian, dorsal and predominantly in brown adipose tissue. These results show that *L. infantum* parasites can be found both in white and brown adipose tissue regardless of their different physiological roles, even though persistence in white adipose tissue is shorter. Mechanisms leading to the elimination of parasites in white adipose tissue over time would be of interest to study.

As mentioned in the results section, parasite burden in adipose tissue was insufficient for bioluminescence detection using the previously generated LUC-*L. infantum* parasites, thus we could not follow-up the infection in vivo. It would be interesting in the future to generate new highly bioluminescent strains allowing detection of few parasites, in order to characterize persistence in adipose tissue, and possibly shed light on other possible persistence sites.

As adipose tissue also contains macrophages, we investigated by qPCR the presence of parasites in an adipocyte-enriched fraction. Because presence of *L. infantum* parasites was demonstrated only in this fraction and not in the macrophage-containing SVF fraction, we analyzed by electron microscopy the infection of adipocytes by *L. infantum* parasites. For that purpose, we used adipocytes differentiated from murine pre-adipocytes isolated from stromal vascular cells. Visualization of electron microscopy data, 48h post infection has shown entrance and presence of a vacuole around *L. infantum* parasites. Interestingly, we could not found vacuoles containing multiple parasites. It would be interesting to perform further experiments to assess whether or not parasites are able to multiply in adipocytes.

Indeed, as it has been shown for *Mycobacterium tuberculosis*^10^, we can hypothesize that within the adipocytes *L. infantum* is in a dormancy state waiting for a better time to exit its host cell. Moreover, transfer of BAT from previously infected BALB/c to naive mice led to development of infection. This indicates that the *L. infantum* parasites present in BAT are infectious parasites, able to infect the liver, spleen and BAT of naive mice. We have shown that, in vitro, *L. infantum* can infect mouse and human adipocytes equally. Furthermore, BAT has been discovered recently in human adults in several anatomical regions^20,21^, thus, we suppose that *L. infantum* can similarly infect humans, as is described for rodents herein.

Another advantage for the parasite to infect adipocytes is that these cells are not professional phagocytes with inherent antimicrobial activities compared to macrophages, which are typically considered to be the host cell reservoir for *Leishmania* parasites. Nevertheless, it has been demonstrated that dermal adipocytes displayed antimicrobial activity^22^. Also, it will be interesting to evaluate this in white, brite and brown adipocytes to tentatively correlate antimicrobial capacity to the variation of persistence duration between these tissues. Our results further suggest that treatments with poor access to adipose tissue would be poorly effective at resolving the infection and would likely be followed by relapses. Further studies will be necessary to determine in humans whether adipocytes could be a reservoir for *L. infantum.* These findings could have an impact on future treatment development, taking into account drug bioavailability in newly found persistence sites to avoid relapses.

## Methods

### Mice and ethics statement

Design and realization of animal experiments follow the ARRIVE guidelines. Female BALB/c mice were purchased from Charles River (France). Mice were maintained and handled according to the regulations of the European Union, the French Ministry of Agriculture and to FELASA (Federation of Laboratory Animal Science Associations) recommendations. Experiments were approved by the ethics committee of the Nice School of Medicine, France (Protocol number: 2017-56).

### Statistical analysis

GraphPad Prism 9 was used for statistical analysis. Significance was determined by analysis of variance (ANOVA) using a Bonferroni correction for multiple comparisons. A p-value < 0.05 was considered significant.

### *L. infantum* culture

*L. infantum* MON-1 (MHOM/FR/94/LPN101), was isolated from a patient with Mediterranean visceral leishmaniasis contracted in the Nice area (South of France). We used this isolate to generate a recombinant *L. infantum*—expressing the Green Fluorescent Protein reporter (GFP-*L. infantum*) and *L. infantum*—expressing the Luciferase reporter (LUC-*L. infantum*)^9^. *L. infantum* promastigotes were routinely grown at 26 °C in Schneider’s Insect Medium (Sigma^®^) supplemented with NaHCO3 0.4 g/L (Janssen chimica^®^), CaCl_2_ 0.6 g/L (Fluka Chemika^®^), Fetal Bovine Serum 10% (Gibco^®^), 10 mL urine pool for 500 mL of medium, Phenol Red 0,1%, Hepes 10 mM pH 7.3, penicillin/streptomycin 1% (Gibco^®^), and l-Glutamine 1% (Gibco^®^).

### Parasite preparation and inoculation in mice

Briefly the promastigote forms were washed three times in PBS, and 2 × 10^8^ parasites were injected by intravenous route in 200 µL of PBS. Control mice were injected with 200 µL of PBS.

### Minced BAT tissue from infected BALB/c

BAT from infected BALB/c mice was sampled and freshly minced using a potter. The minced infected BAT was injected by intraperitoneal route in naive BALB/c mice.

### Separation of an adipocyte-enriched fraction and a stromal vascular fraction (SVF)

Briefly BAT and WAT were minced and then digested for 45 min at 37 °C in collagenase type 2. The tissue digest was passed through 250 μm nylon sheets. Floating adipocytes were separated from the SVF after decantation. The floating fraction corresponding to the adipocyte-enriched fraction was carefully removed.

### Quantification of parasites by quantitative PCR

Each sample of Adipose tissue, liver and spleen (25 mg) was put in a sterile tube of Lysing Kits (Precellys^®^), and then homogenized by Precellys^®^ (2 × 30 s, with a break of 15 s) in lysis buffer of the Qiagen Kit QIAmp DNA Mini Kit^®^. DNA extraction was conducted according to the recommendations of Qiagen^®^. The extracts were kept at − 20 °C for conservation. Quantitative PCR was implemented for detection and quantification of *L. infantum* targeting minicircle kinetoplast DNA (kDNA). Primers and probes previously described by Mary et al.^5^ containing 20 pmol of each forward (5′-CTTTTCTGGTCCTCCGGGTAGG-3′) and reverse (5′-CCACCCGGCCCTATTTTACACCAA-3′) primer and 3.33 pmol TaqMan probe (FAM-TTTTCGCAGAACGCCCCTACCCGC-TAMRA) were used for *Leishmania* screening and quantification^5^. The assays were performed with a final volume of 10 µL, including the 2.5 µL DNA sample. A standard curve was obtained from the primary DNA extraction source of 2.5 × 10^7^ parasites and diluted serially at a 1/10 rate, which corresponded to 50,000 to 0.05 parasites in 2.5 µL. The PCR program was implemented with two temperature steps of 95 °C and 60 °C for 40 cycles. The standard curve and a pair of negative controls were used for each assay.

### Histology and microscopic observation

Organs were fixed in 4% PFA (ParaFormAldehyde). Samples were embedded in paraffin automatically with the spin tissue processor STP120 (ThermoFisher^®^). The tissue processor STP120 uses alcohol to remove water from tissues and replace it with a medium that allows sectioning of tissue. Thin sections (2.5 µm) were cut with a Microtome Microme HM340E (Leica BIOSYSTEMS^®^). Sections were deparaffinized by immersing 3× in xylene, rehydrated by successive immersion in ethanol solutions of different percentages (100%, 95%, 70%, 50%) and in water. Unmasking was conducted boiling in 10 mM Sodium Citrate buffer (pH 6.0). Endogenous peroxidase activity was blocked by a solution of 3.0% hydrogen peroxide. The immuno-histochemistry labeling consisted of a primary human antibody directed against *Leishmania*. This antibody was recognized by an anti-human goat antibody, which was recognized by biotinylated anti-goat, the signal was amplified and revealed by Streptavidin-HRP. The brightfield microscope was an Eclipse Ci upright stand (Nikon, Japan), using objectives 20× dry NA 0.40. Acquisitions were done with a DS—Ri 1 camera (Nikon, Japan).

### Mouse primary pre-adipocyte purification and differentiation

The method for generating white, brite and brown adipocytes from stromal vascular fraction (SVF) cells was adapted from a previous publication^23^. Briefly, fat deposits were sampled, minced and then digested for 45 min at 37 °C in DMEM (Lonza, BE12–707F) containing 2 mg/mL collagenase A (Roche Diagnostics, 11088793011) and 20 mg/mL BSA (Sigma-Aldrich Chemie Gmbh, A7030). The digestion was successively filtrated through 250, 100 and 27 μm nylon sheets, and finally centrifuged at 500×*g* for 5 min. The pellet containing the SVF was cleared from red blood cells using specific buffer (Sigma) before being plated and maintained in DMEM containing 10% (v/v) fetal calf serum (FCS) until confluence. Differentiation was induced by supplementation with 1 μM dexamethasone (Sigma-Aldrich Chemie Gmbh, D4902), 0.5 mM isobutylmethylxanthine (Sigma-Aldrich Chemie Gmbh, I5879) and 860 nM insulin (Invitrogen, 12585014) for 2 days. Cells were then maintained for 7–10 days in presence of 100 nM insulin for white adipogenesis or a mixture containing 100 nM insulin, 1 μM rosiglitazone (BertinPharma, 71740) and 0.2 nM triiodothyronine (Sigma-Aldrich Chemie Gmbh, T6397) for brown or brite adipogenesis.

### Differentiation and generation of 3T3 adipocytes

3T3-L1 fibroblasts were grown at 7% CO2 and 37 °C on coverslips in 35 mm dishes in DMEM, 25 mm glucose, and 10% calf serum, and 1% Penicillin–Streptomycin, and induced to differentiate in adipocytes. Briefly, 2 days after confluence, medium was changed for DMEM, 25 mM glucose, 1% Penicillin–Streptomycin, and 10% fetal calf serum (FCS) supplemented with isobutylmethylxanthine (0.25 mM), dexamethasone (0.25 µM), insulin (5 µg/mL), and pioglitazone (10 µM). The medium was removed after 2 days and replaced with DMEM, 25 mM glucose, 1% Penicillin–Streptomycin, and 10% FCS supplemented with insulin (5 µg/mL) and pioglitazone (10 µM) for 2 days. Then the 3T3-L1 adipocytes were fed every 2 days with DMEM, 25 mM glucose, 1% Penicillin–Streptomycin, and 10% FCS.

### Differentiation and generation of macrophages

For *BMDM* (Bone Marrow Derived Macrophage), mouse femurs were removed and purified from the surrounding muscles and connective tissue. Under sterile conditions, the bone marrow was flushed by pressure after needle penetration in epiphyses with BMDM medium containing RPMI and decomplemented FBS 10% and gentamycin 0.001%. The cells were centrifuged (400×*g*, 5 min) and resuspended in BMDM medium supplemented with M-CSF 10 ng/mL. Cells were seeded at 5 × 10^5^ cells/well.

Blood monocytes were isolated from human healthy blood samples (leukoplatelet layer, Etablissement Français du Sang) using EasySep™ Human Monocyte Enrichment Kit (STEMCELL Technologies) according to manufacturer’s instructions. Macrophage differentiation was induced by human M-CSF (Peprotech, 100 pg/mL, 5 days).

### Human adipocytes differentiation

Human adipose tissue primary progenitor cells were from a previous study^19^ and differentiated as follow. Cells were cultivated in DMEM containing 10% FCS until confluence. When the cells reached confluence, they were induced to differentiate for 3 days in DMEM/Ham’s F12 (1:1) media supplemented with 10 μg/mL transferrin, 10 nM insulin, 0.2 nM triiodothyronine, 1 μM dexamethasone and 500 μM isobutyl-methylxanthine. The cells were next differentiated into white adipocytes using a media supplemented with 10 μg/mL transferrin, 10 nM insulin, 0.2 nM triiodothyronine or into brite adipocytes in the same media supplemented with 100 nM rosiglitazone.

### Isolation and analysis of RNA

Total RNA was extracted using a TRI-Reagent kit (Euromedex) according to the manufacturer’s instructions. Reverse transcription-polymerase chain reaction (RT-PCR) was performed using M-MLV-RT (Promega). SYBR qPCR premix Ex Taq II from Takara (Ozyme) was used for quantitative PCR (qPCR), and assays were run on a StepOne Plus ABI real-time PCR instrument (PerkinElmer Life and Analytical Sciences). The expression of selected genes was normalized to that of the 36B4 (RPLP0, Ribosomal Protein Lateral Stalk Subunit P0) and TBP (TATA-box protein) housekeeping genes and then quantified using the comparative-ΔCt method. Primer sequences are available upon request.

### In vitro infection

The BMDM and adipocytes of pre-adipocyte murine origin were infected after 7 days of differentiation with a ratio of 10:1 GFP-*L. infantum* per cell. Human adipocytes were infected after 14 days of differentiation with a ratio of 10:1 GFP-*L. infantum* per cell. 3T3 cells were infected 8 days after their differentiation with a ratio of 10:1 GFP-*L. infantum* per cell.

### Epifluorescence and confocal microscopy

For epifluorescence acquisition, we used the EVOS FL microscope (AMF-4302-EU; Labtech, France), using the 10× dry Ph and 20× dry FL objectives. Acquisitions were done with a Sony ICK285AL monochrome CCD, 2/3′′ 1360 × 1024, 1.4 Megapixel camera (Labtech, France). For confocal microscopy, F-actin was labeled with phalloidin-TRITC (red), and the nuclei were labeled with DAPI (blue). *Leishmania*-GFP parasite are green. The fluorescent signals were analyzed with a Nikon confocal microscope using a × 60 magnification lens.

### Electron microscopy

For ultrastructural analysis, cells were fixed in 1.6% glutaraldehyde in 0.1 M phosphate buffer (pH 7.4) at 4 °C, rinsed in 0.1 mol/L cacodylate buffer, and fixed for 1 h in 1% osmium tetroxide and 1% potassium ferrocyanide in 0.1 mol/L cacodylate buffer to enhance the staining of membranes. Cells were rinsed in cold distilled water, quickly dehydrated in cold ethanol, and lastly embedded in epoxy resin. Contrasted ultrathin sections (70 nm) were analyzed under a JEOL 1400 transmission electron microscope (EM) mounted with a Morada Olympus charge-coupled device camera.

## Supplementary Information

Supplementary Legends.

Supplementary Figure S1.

Supplementary Figure S2.

Supplementary Figure S3.

Supplementary Figure S4.

Supplementary Figure S5.

Supplementary Figure S6.
