# Supplementary material for: Identification of adipocytes as target cells for Leishmania infantum parasites
Source: Sci Rep. 2021 Oct 28;11:21275. doi: 10.1038/s41598-021-00443-y (PMC8553825; doi:10.1038/s41598-021-00443-y)
Supplement: Supplementary file 1 — Supplementary Legends. [file 41598_2021_443_MOESM1_ESM.docx]

**Supplemental figures legend**

**Figure S1.** Parasite burden per milligram of spleen and liver at 6 (A), 10 (B) and 31 weeks post infection (C) by qPCR. BALB/c mice were infected intravenously with 2x10^8^ LUC-*Leishmania infantum*. * = p<0,05.

**Figure S2.** Parasite burden per 100 milligram of tissue in organs of naive mice transplanted with BALB/c-infected brown adipose tissue (AT). DNA was extracted in a 100 µL volume and the presence of *L. infantum* DNA was determined by qPCR using a 2.5 µL DNA extract. * = p<0,05.

**Figure S3.** *In vitro* GFP-*L. infantum* infection of BALB/C adipocytes. The BMDM and adipocytes of pre-adipocytes murine origin were infected after 7 days of differentiation with 10 GFP-*Leishmania*/cells. Images were acquired with EVOS Fl software from ThermoFisher Scientific (<https://www.thermofisher.com>). Images were merged with ImageJ bundled with Java 1.8.0_172 (<https://imagej.nih.gov/ij/>). Images were assembled with Adobe Photoshop 2020 (<https://www.adobe.com/>).

**Figure S4.** Fluorescence microscopy of different subsets of human adipocytes infected *In vitro* with GFP-*L. infantum*. Human adipocytes were untreated (Control) or infected after 14 days of differentiation with 10 GFP-*Leishmania*/cells. Images were acquired with EVOS Fl software from ThermoFisher Scientific (<https://www.thermofisher.com>). Images were merged with ImageJ bundled with Java 1.8.0_172 (<https://imagej.nih.gov/ij/>). Images were assembled with Adobe Photoshop 2020 (<https://www.adobe.com/>).

**Figure S5. A.** mRNA expression of PLN 1 and CD11b determined by RT-qPCR on lysates of murine *in vitro* differentiated adipocyte as compared to BMDM. **B.** mRNA expression of CXCL 10, mrc1, UCP1 and Perilipin 1 determined by RT-qPCR on lysates of human *in vitro* differentiated adipocyte as compared to PBMC. Histograms represent mean + sem of 4 lysates.

**Figure S6.** *In vitro* GFP-*L. infantum* infection of 3T3 adipocytes. The 3T3 adipocytes were infected after 8 days of differentiation with 10 GFP-*Leishmania*/cell. Red: phallloidin-Txred, Blue: Dapi, Green: GFP-leish. Images were acquired with Nikon Confocal A1R software (NIS-Elements Confocal) from Nikon (https://www.microscope.healthcare.nikon.com). Images were merged with ImageJ bundled with Java 1.8.0_172 (<https://imagej.nih.gov/ij/>). Images were assembled with Adobe Photoshop 2020 (<https://www.adobe.com/>).
