# Supplementary figures and images for: Identification of adipocytes as target cells for Leishmania infantum parasites
Source: Sci Rep. 2021 Oct 28;11:21275. doi: 10.1038/s41598-021-00443-y (PMC8553825; doi:10.1038/s41598-021-00443-y)

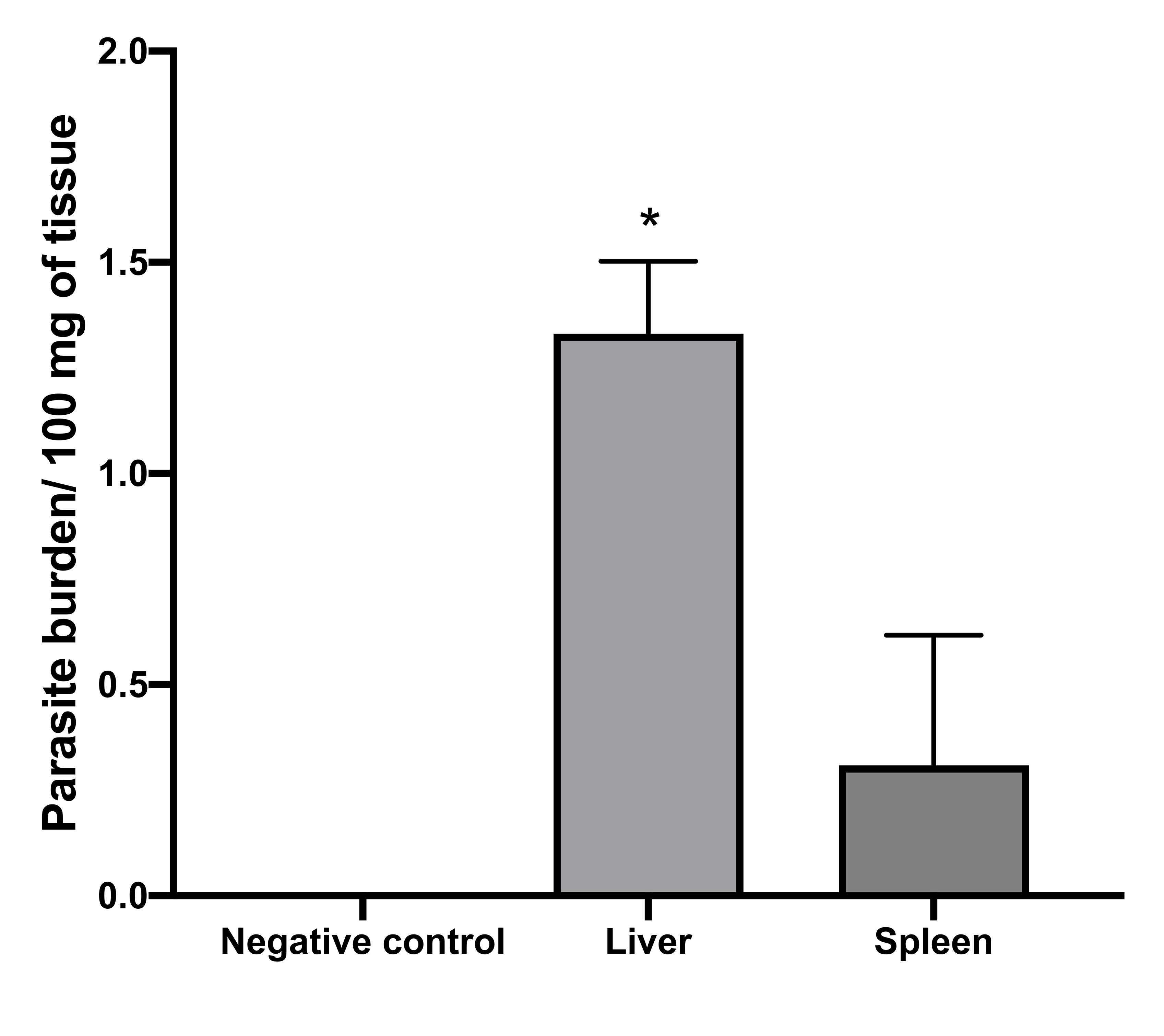

Supplement: Supplementary file 3 — Supplementary Figure S2. [file 41598_2021_443_MOESM3_ESM.tif]

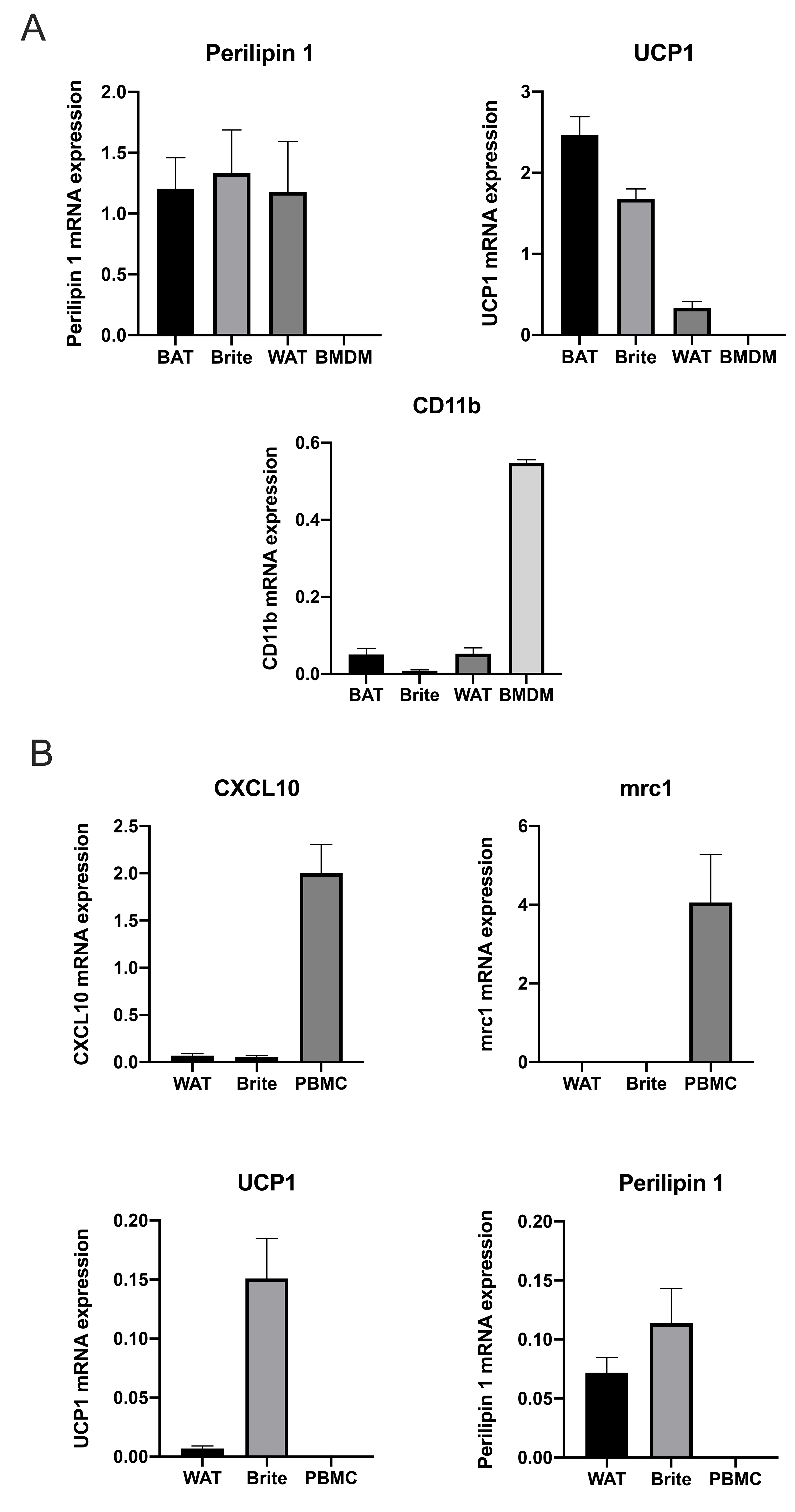

Supplement: Supplementary file 6 — Supplementary Figure S5. [file 41598_2021_443_MOESM6_ESM.tif]

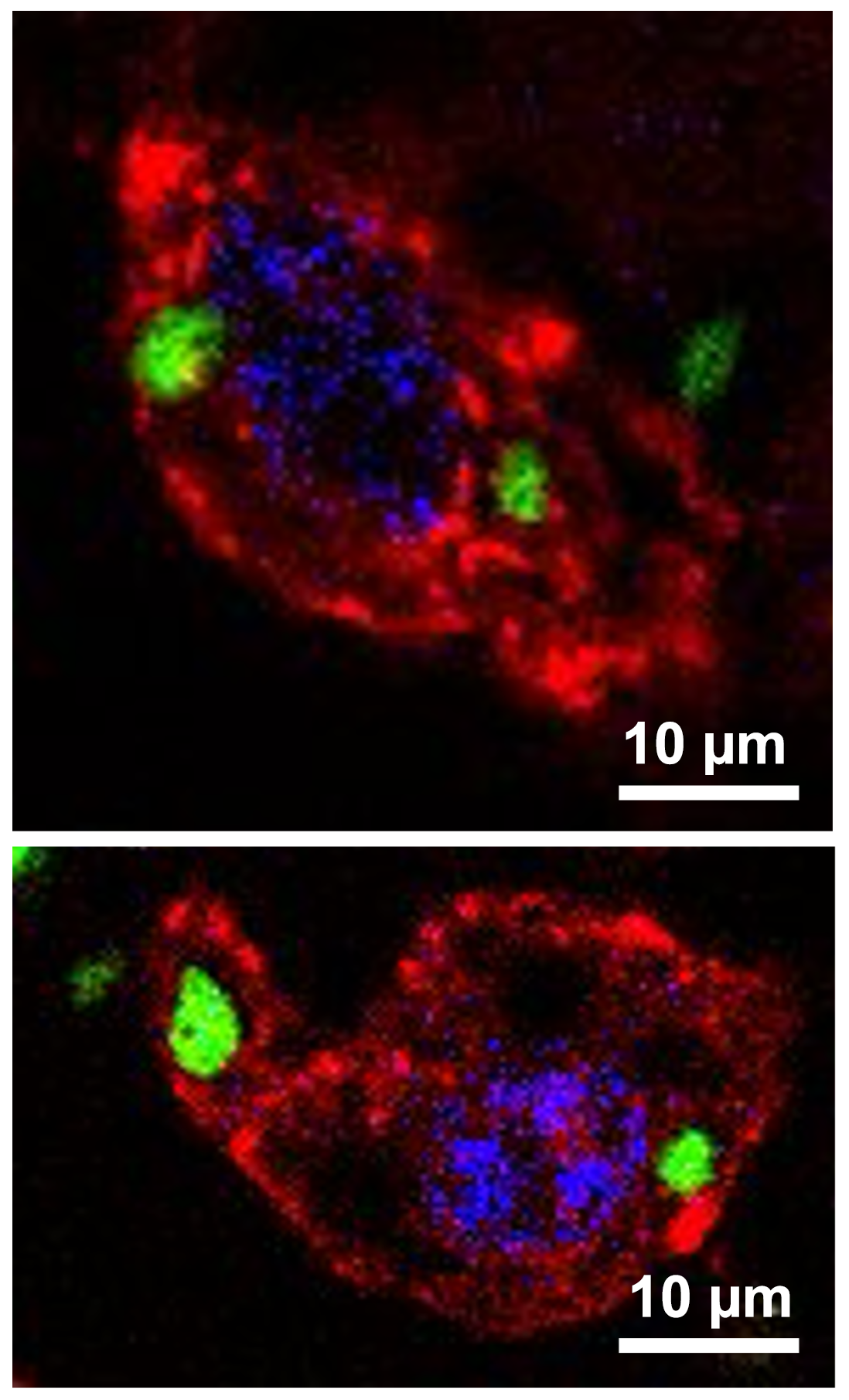

Supplement: Supplementary file 7 — Supplementary Figure S6. [file 41598_2021_443_MOESM7_ESM.tiff]
